# Logsum Using Garbled Circuits

**DOI:** 10.1371/journal.pone.0122236

**Published:** 2015-03-26

**Authors:** José Portêlo, Bhiksha Raj, Isabel Trancoso

**Affiliations:** 1 INESC-ID, Lisbon, Portugal; 2 Instituto Superior Técnico, Universidade de Lisboa, Lisbon, Portugal; 3 Language Technologies Institute, Carnegie Mellon University, Pittsburgh, Pennsylvania, United States of America; University of Catania, ITALY

## Abstract

Secure multiparty computation allows for a set of users to evaluate a particular function over their inputs without revealing the information they possess to each other. Theoretically, this can be achieved using fully homomorphic encryption systems, but so far they remain in the realm of computational impracticability. An alternative is to consider secure function evaluation using homomorphic public-key cryptosystems or Garbled Circuits, the latter being a popular trend in recent times due to important breakthroughs. We propose a technique for computing the logsum operation using Garbled Circuits. This technique relies on replacing the logsum operation with an equivalent piecewise linear approximation, taking advantage of recent advances in efficient methods for both designing and implementing Garbled Circuits. We elaborate on how all the required blocks should be assembled in order to obtain small errors regarding the original logsum operation and very fast execution times.

## Introduction

An increasing number of server-based applications perform tasks such as classification, processing and analysis of user data. Often, these data are *private*, and should not be exposed to the server. Likewise, the server’s inputs to these computations are also private and may not be exposed to the user. Hence, it becomes necessary to perform the computations in a *privacy preserving* manner, such that the user’s data and the server’s inputs are not revealed to one another, while yet ensuring that the appropriate party gets the correct result from the computations.

Stated in abstract terms, a user Alice possesses data **x** (e.g. email, a biometric measurement, or a voice sample). System Bob is capable of computing a function *f*(**x**;*θ*) with parameter *θ* (e.g. a spam filter or a biometric classifier). Alice desires to obtain *f*(**x**;*θ*) from Bob; however, she is unwilling to expose **x** to him. Bob too is unwilling to expose *θ* to Alice. The challenge of privacy-preserving computation is to enable Alice to obtain *f*(**x**;*θ*) such that Bob learns nothing of **x** and Alice learns nothing more of *θ* besides what she may glean from *f*(**x**;*θ*) itself.

In principle, such transactions could be achieved using fully homomorphic encryption [1], which permits computation to be performed on encrypted data. However, practical fully homomorphic encryption schemes remain computationally impractical in spite of recent advances [2, 3].

Instead, Alice and Bob must achieve their goals through secure function evaluation (SFE) [4, 5]. SFE involves repeated exchange of partial information between Alice and Bob using protocols which mask the exchanged values using a combination of techniques such as public-key encryption [6, 7], oblivious transfer (OT) [8], etc., until the appropriate party gets the desired result.

Arguably the most popular approach to SFE is to cast the function to be evaluated as a *garbled* Boolean circuit (GC) [4, 9, 10]. Like logic circuits, GCs are composed of logic gates, with each gate represented by a truth table. However, while in regular logic circuits the inputs and outputs of each truth table are binary values (0/1), in GCs each possible entry of the truth table is an encrypted value which can only be “unlocked” by a input-specific key pair. The entries, in turn, too are keys which may be used to unlock other gates downstream the circuit.

Although in principle any function could be computed in this manner, practical realizations face computational challenges. Evaluation of a GC requires exchange of information through OT and repeated decryption of gate outputs, both of which are highly expensive operations. Ideally the circuit itself must be designed to minimize this overhead.

Traditionally, circuit optimization has largely been viewed as the problem of minimizing the number of gates in the circuit. The problem of finding the smallest circuit to perform a given Boolean function is known to be intractable in general [11], although a number of effective algorithms have nevertheless been proposed [12–15].

In the case of GCs, we must also consider other factors. Not all gates are equally expensive to compute: gates such as NOT and XOR do not require explicit encryption in a GC and are orders of magnitude cheaper to compute than other gates, and must preferentially be used. Elsewhere, where feasible, complex operations may need to be replaced with cheaper approximations or or encrypted table lookups. GCs do not support looping operations. In addition, in situations where the underlying computation is floating-point, the effect of the loss of resolution from fixed-point implementation must also be considered.

The design of Garbled Circuits is thus a complex problem. A number of toolkits have been developed to support the conversion of algorithmic circuits to their garbled forms [16–19]. Typically, they include several optimized garbled components for “primitives”, such as multiplication, maximum-value computation, vector inner-products, etc., which may be employed in the design of larger circuits. All of these primitives are *simple*, in the sense that they embody linear arithmetic computation over one or more inputs.

In this paper we develop a GC for a very important complex non-linear primitive, the *logsum* operation, a key building block in many statistical and signal processing computations. We build upon previous work [20, 21]. In [20] the authors use a GC to compute the logarithm as an integer operation, i.e., for input *a* > 0, the logarithm is computed as *b* = ⌊log_2_ *a*⌋+1. Because *b* is always an integer approximating the real value of the logarithm, this technique would lead to large errors if it were used for computing the logsum operation. In [21] the authors use a homomorphic encryption system to compute the logsum by successively performing secure protocols for exponentiation and logarithms. While this technique has reduced error with appropriate pre-scaling of the inputs, the encryption imposes large computational overhead and performing all the required protocols requires substantial amounts of time. In order to avoid the large computation errors or large execution times of the previous approaches, our design casts the logsum operation as a combination of simple operations such as table lookups, additions and multiplications, arranged to minimize the loss of resolution in the output while simultaneously retaining efficiency through the use of low-cost computational units, such that the actual garbled computation may be performed in reasonable time.

This paper starts with necessarily brief overviews of the different operations involved in logsum computations, and the basic idea underlying Garbled Circuit protocols. We propose a new method for computing the logsum operation using GCs, starting with an array of just two values, and later generalizing it to any number of input values. The performance of the proposed method is then analyzed for two different GC toolkits in terms of execution time for different numbers of input values, different lengths (in bits) of these values, and different operation parameters. The paper ends with some concluding remarks.

## Methods

### Logsum operation

In many signal processing and pattern classification problems it is necessary to compute the occurrence probability of an event *X*. The probability models employed are commonly mixture models of the form $P(X)=\sum_{i=1}^{N}P(X,Y_{i})$. In order to avoid underflow/overflow problems, the actual computations are usually performed with *log* probabilities. Rather than computing *P*(*X*), one works with log*P*(*X*), which is in turn expressed as in terms of the logarithm of the component probabilities as

$$\begin{array}{c} z\left(m\right)=\log\left(\sum_{i=1}^{N}\exp\left(m_{i}\right)\right), \end{array}$$ (1)

where *z*(*m*) = log(*P*(*X*)), and *m*_*i*_ = log(*P*(*X*,*Y*_*i*_)). The equation above is a *logsum* operation. To further avoid overflow/underflow problems that arise when all the *m*_*i*_ terms become too large in magnitude, Equation 1 is usually further recast as

$$\begin{array}{c} z\left(m\right)=m_{X}+\log\left(\sum_{i=1}^{N}\exp\left(m_{i}-m_{X}\right)\right), \end{array}$$ (2)

with *m*_*X*_ = max_*i*_ *m*_*i*_.

Logsum operations are found in a variety of signal processing and statistical computation tasks, such as sparse signal recovery [22], matrix recovery [23], and in the evaluation of a large variety of statistical models such as latent Dirichlet allocation [24] and Gaussian mixture models [25]. Thus, the logsum operation may in fact be considered one of the key building blocks in statistical and signal processing computations.

### Garbled Circuits

**Fig 1:**
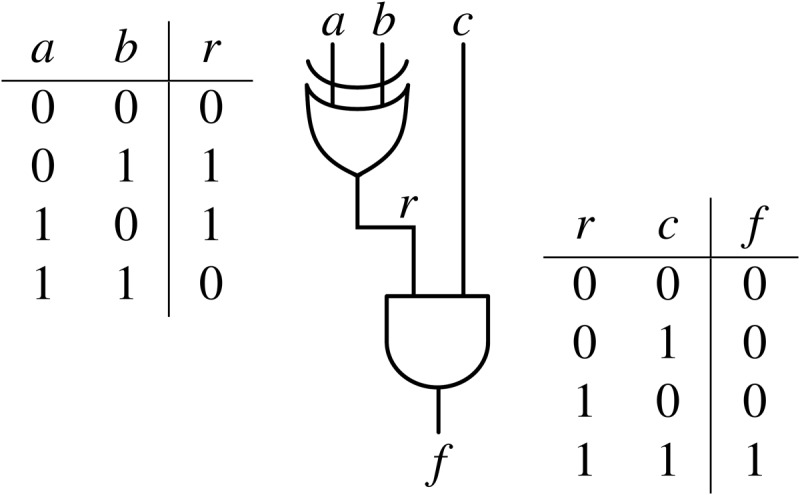
Example of a logic circuit and corresponding truth tables. The circuit implements an XOR gate (*r* = *a*⊕*b*) followed by an AND gate (*f* = *r*∧*c*). The binary truth tables for the XOR and AND gates are presented on the left hand side and right hand side of the circuit, respectively.

Garbled Circuits are best explained with an example. Bob has Boolean input *a* and Alice has inputs *b* and *c*. They wish to compute the function *f*(*a*,*b*,*c*) = (*a*⊕*b*)∧*c*. The logic circuit for *f*(*a*,*b*,*c*) and the truth tables for the gates in it are shown in Fig. 1. However, Bob does not want to reveal *a* to Alice, and Alice does not want to reveal *b* or *c* to Bob. In order to still be able to compute *f*(*a*,*b*,*c*), they must hence *garble* the circuit.

Bob generates the logic circuit implementing *f*(⋅). Thereafter, for each input and intermediate value (*a*, *b*, *c*, *r* in the example), Bob generates two private keys, one for each bit value, totaling eight keys in our example: $K_{a}^{0/1}$, $K_{b}^{0/1}$, $K_{c}^{0/1}$, $K_{r}^{0/1}$. For gates that generate intermediate values (*r* = *a*⊕*b* in our example), he replaces the outputs of the truth table with the encryption of the key corresponding to the output, performed with the keys corresponding to the inputs. For example, for *a* = 0, *b* = 1 the output is *r* = 1; the corresponding input keys are $K_{a}^{0}$, $K_{b}^{1}$ and the encrypted output is ${ℰ}_{K_{a}^{0}}({ℰ}_{K_{b}^{1}}(K_{r}^{1}))$. For the output gate (*f* = *r*∧*c* in our example) he encrypts the output value itself. The garbled values for our example are presented in Table 1.

**Table 1:** Example of garbled truth tables.

*a*	*b*	*r*
$K_{a}^{0}$	$K_{b}^{0}$	${ℰ}_{K_{a}^{0}}({ℰ}_{K_{b}^{0}}(K_{r}^{0}))$
$K_{a}^{0}$	$K_{b}^{1}$	${ℰ}_{K_{a}^{0}}({ℰ}_{K_{b}^{1}}(K_{r}^{1}))$
$K_{a}^{1}$	$K_{b}^{0}$	${ℰ}_{K_{a}^{1}}({ℰ}_{K_{b}^{0}}(K_{r}^{1}))$
$K_{a}^{1}$	$K_{b}^{1}$	${ℰ}_{K_{a}^{1}}({ℰ}_{K_{b}^{1}}(K_{r}^{0}))$

*r*	*c*	*f*
$K_{r}^{0}$	$K_{c}^{0}$	${ℰ}_{K_{r}^{0}}({ℰ}_{K_{c}^{0}}(0))$
$K_{r}^{0}$	$K_{c}^{1}$	${ℰ}_{K_{r}^{0}}({ℰ}_{K_{c}^{1}}(0))$
$K_{r}^{1}$	$K_{c}^{0}$	${ℰ}_{K_{r}^{1}}({ℰ}_{K_{c}^{0}}(0))$
$K_{r}^{1}$	$K_{c}^{1}$	${ℰ}_{K_{r}^{1}}({ℰ}_{K_{c}^{1}}(1))$

[Left] Garbled table for the XOR gate (*r* = *a*⊕*b*). The garbled values of the keys $K_{r}^{0/1}$ associated with output *r*, ${ℰ}_{K_{a}^{0/1}}({ℰ}_{K_{b}^{0/1}}(K_{r}^{0/1}))$, are encrypted using the keys $K_{a}^{0/1}$ and $K_{b}^{0/1}$ associated with inputs *a* and *b*, respectively. [Right] Garbled table for the AND gate (*f* = *r*∧*c*). The garbled values of output *f*, ${ℰ}_{K_{r}^{0/1}}({ℰ}_{K_{c}^{0/1}}(0/1))$, are encrypted using the keys $K_{r}^{0/1}$ and $K_{c}^{0/1}$ associated with inputs *r* and *c*, respectively.

To compute the function Bob transmits the keys corresponding to his bit choice for his inputs $K_{a}^{?}$ to Alice. Alice recovers the keys corresponding to the bit choice for her inputs $K_{b}^{?}$, $K_{c}^{?}$, as well as the truth tables for the gates of the circuit from Bob using oblivious transfer [8]. Because of the properties of OT, Bob does not learn either the value of Alice’s bits or the portion of the truth table she actually needs to perform her computations. To evaluate the circuit Alice successively evaluates each of the gates in the circuit. The nature of the computation is such that for each gate, Alice will possess two keys, one for each input. She decrypts all four encrypted output in the truth table for the gate, using her two keys. The keys will be inappropriate for three of the four outputs; hence Alice can only correctly decipher one of the four encrypted values, which will be a key for the next gate. After repeating this process for all gates, she finally obtains the desired value *f*. Alice and Bob never learn each others’ inputs.

For a long time it was believed that GCs were of purely theoretical interest, but many advances have made GC much more efficient and practical to use. For instance, all the OTs for transferring the input ciphers may be pre-computed [26], meaning that all the computationally expensive operations regarding the OT are performed offline, and only an additional yet simple operation per OT is performed while evaluating the GC. Furthermore, all the OT may be computed efficiently by using shorter ciphers [27, 28], which can be implemented using elliptic curve cryptography [29]. In the gate decryption phase, the point and permute technique [16] may be used to reduce the total number of required decryptions from 4 to 1 by associating a permutation bit to the input ciphers chosen using OT. Finally, all the XOR gates of a GC may be evaluated for “free” [30], i.e., without significant computational cost, as the XOR gate does not need a garbled table and its evaluation consists of XOR-ing its garbled input values. NOT gates are similarly “free” as well. Custom designing circuit operations to preferentially utilize these gates will result in faster evaluation of the resulting Garbled Circuit [31].

On top of these implementation tricks, other useful properties of GC have been found. For example, it is possible to reuse the same GC several times under some conditions [32] by using functional encryption [33, 34], which means that if the same operation must be computed several times we only need a single circuit instead of one circuit for each time we want to compute that function. Moreover, several privacy and security guarantees regarding GC have been proven [9, 35–37].

In implementing the basic operations using GC, some issues different from the ones faced when using hardware circuits arise. Decision making operations such as comparisons (greater/lesser-than, maximum/minimum) and multiplexing can be implemented similarly to digital circuits. However, branching (if-then-else) and cycles/recursion (for loop, do-while) should be avoided. The first requires building and evaluating circuits for all possibilities in order to hide which one was actually chosen. The second requires explicitly expanding the cycles, one circuit per iteration, which may result in a huge intractable circuit. Another problem arises when computing non-linear functions, since the usual trick of computing the initial elements of the corresponding Taylor series is impractical to implement using GCs. An efficient work-around is to replace these operations with piecewise linear approximations (PLA), whose coefficients can be stored in look-up tables. An additional restriction exists on the number representation of the ciphered values. Operations on floating-point values usually require some normalization on them before they are performed, introducing an additional and unnecessary computational cost. To counter this, a fixed-point representation is preferred, despite a possible loss of precision in representing those same values.

Finally, given the wide-spread popularity of GCs, a huge variety of software implementations of all the required protocols and encryption systems are available. One of the first practical implementations of GC was Fairplay [16], and since then a number of different implementations have been developed in order to further improve their efficiency and practicality [17–19].

### Logsum operation using Garbled Circuits

We now show how to perform the computation of the logsum of an array of values using GC. We start by describing how the logsum operation can be computed using a piecewise linear approximation, then we describe the full circuit for the simple situation where there are only two input values, and finally we generalize it to any number of input values.

#### Piecewise linear approximation

Many algorithms rely on evaluating non-linear functions in order to compute an output. These functions are normally replaced by the corresponding Taylor series, whose terms are computed until the error between iterations falls below a pre-determined threshold. However, when limited computational resources are available, the non-linear function is often be replaced by a PLA. A method for computing piecewise linear approximations using GCs has already been proposed [38], but it only focuses on a 1^*st*^-order approach (ramp quantization (RQ)), discarding a 0^*th*^-order approach (step quantization (SQ)). It is nevertheless instructive to evaluate both options in the context of GCs, where computation is at a premium. Although it is expected that the error obtained with the SQ approach should be larger than the one obtained with the RQ approach, the computational benefits of the lower-order approximation can outweigh the increased error from loss of resolution.

A standard way to implement a PLA is to split the domain of the input function *f*(⋅) into *k* intervals, and then compute linear approximations *f*(⋅) for each interval, adjusting all the interval limits *t*_*i*_ and linearization parameters *n*_1,*i*_ and *n*_2,*i*_ in order to obtain the minimum error possible regarding *f*(⋅). These parameters are then placed in the form of a look-up table such as the one presented in Table 2.

**Table 2:** Look-up table with **k** entries for the piecewise linear approximation.

*m*:*t*	*f*(*m*):*n*_1_⋅*m*+*n*_2_	
*m* ≤ *t*_1_	*n*_1,1_	*n*_2,1_
*t*_1_ < *m* ≤ *t*_2_	*n*_1,2_	*n*_2,2_
…	…	…
*t*_*k*−2_ < *m* ≤ *t*_*k*−1_	*n*_1,*k*−1_	*n*_2,*k*−1_
*t*_*k*−1_ < *m*	*n*_1,*k*_	*n*_2,*k*_

Note that the domain of the *i* ^*th*^ interval is given by ]*t*_*i*−1_,*t*_*i*_].

For obtaining the correct parameter pair (*n*_1,*i*_,*n*_2,*i*_) for each value of *m*, it is necessary to compare *m* with all the *t* in order to find an index *j* such that *t*_*j*_ < *m* < *t*_*j*+1_, allowing for computing *f*_PLA_(*m*) = *n*_1,*j*+1_⋅*m*+*n*_2,*j*+1_ ≈ *f*(*m*), an approximation of the desired value. In the situations where *m* < *t*_1_ or *m* > *t*_*k*−1_, the approximation of the desired value is obtained by computing *f*_PLA_(*m*) = *n*_1,1_⋅*m*+*n*_2,1_ or *f*_PLA_(*m*) = *n*_1,*k*_⋅*m*+*n*_2,*k*_, respectively. This last scenario is not unlikely, since many functions have an unbounded domain, meaning that it is necessary to force artificial bounds when optimizing the locations of *t*. Therefore, special care is required while computing n_*,1_ and n_*,*k*_, so that they do not provide increasingly erroneous outputs as the difference between *m* and *t*_1_ or *t*_*k*−1_ increases.

#### Higher order approximations

We did not consider any higher order polynomial approximations for the logsum operation for a variety of reasons. Let *d* be the degree of the approximation polynomial for the logsum operation. Preliminary experiments showed that although there was some small improvement in terms of the obtained error as *d* increased, the improvement rate decreased sharply. In particular, for *d* ≥ 3 (cubic and higher order approximations) the error obtained is almost the same as for the situation where *d* = 2 (quadratic approximation). Also, increasing *d* leads to a quadratic increase in the number of required multiplications for evaluating the look-up table. Even if *d* = 2 is considered, this would require computing three multiplications instead of just one. Since the multiplication is by far the most computationally demanding operation in our GC approach, increasing *d* would lead to a significant and undesirable increase in the overall execution time. Finally, and most importantly, increasing *d* leads to having to perform several multiplications in cascade, which, given a GC approach, causes an exponential increase of the number of bits required to represent the output.

#### Logsum of *N* = 2 values

Recalling Equation 2, the logsum of an array of numbers can be written as

$$\begin{array}{c} z\left(m\right)=m_{X}+\log\left(\sum_{i=1}^{N}\exp\left(m_{i}-m_{X}\right)\right), \end{array}$$ (3)

where *m*_*X*_ = max_*i*_ *m*_*i*_. For the simplest case where *N* = 2, this equation can be simplified to

$$\begin{array}{c} z\left(m\right)=m_{X}+\log\left(1+\exp(m_{N}-m_{X})\right), \end{array}$$ (4)

with *m*_*X*_ = max(*m*_1_,*m*_2_), *m*_*N*_ = min(*m*_1_,*m*_2_). After performing PLA, the approximation for the logsum operation is given by

$$\begin{array}{c} z\left(m\right)\approx m_{X}+\left(n_{1}\cdot \left(m_{N}-m_{X}\right)+n_{2}\right). \end{array}$$ (5)

**Fig 2:**
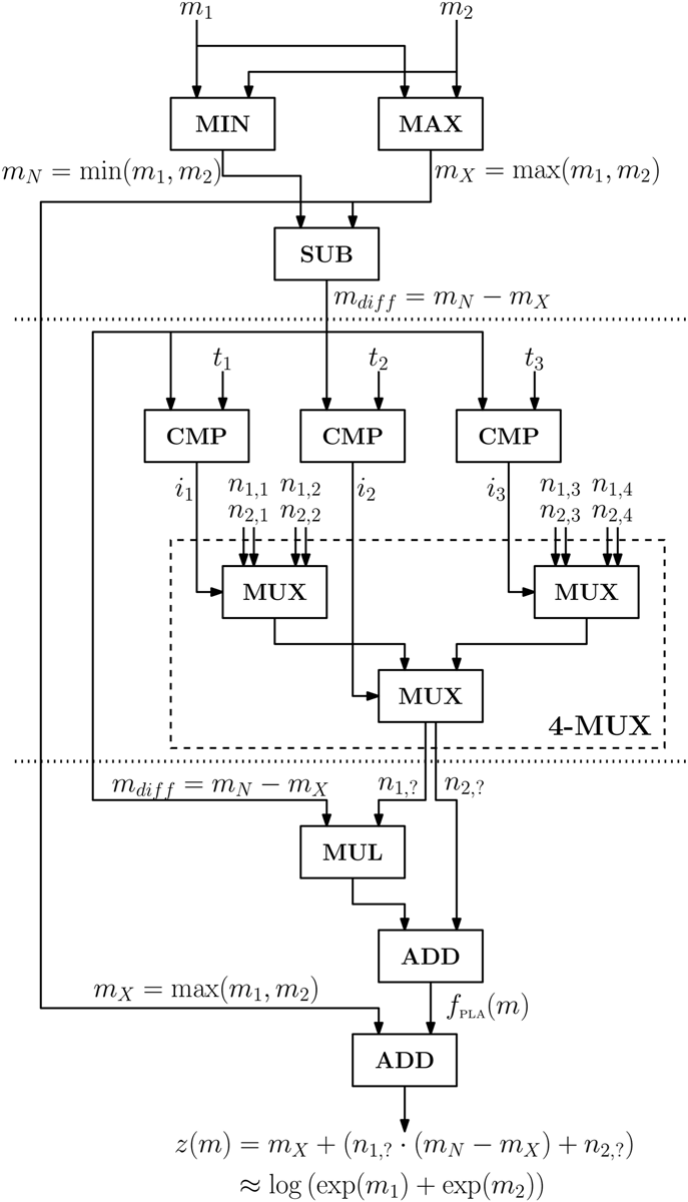
Circuit diagram for the logsum operation with **N = 2** inputs and **k = 4** look-up table entries. There are three major blocks in this diagram, separated from each other by horizontal dotted lines. In each block, the individual boxes correspond to a simple operation. The first block contains the computation of all the necessary elements for obtaining the logsum *z*(*m*), namely *m*_*X*_, *m*_*N*_ and *m*_*diff*_ = *m*_*N*_−*m*_*X*_, by means of computing a minimum (MIN), a maximum (MAX) and a subtraction (SUB), respectively. The second block contains the circuit for obtaining the parameters for the PLA of the logsum operation. It starts by performing comparisons (CMP) between *m*_*diff*_ and the look-up table entries *t*, then the corresponding decisions *i* are used as control inputs to the hierarchical multiplexer (4-MUX), and finally the unknown piecewise linear approximation parameters *n*_1,?_ and *n*_2,?_ are outputted. The third block performs the linear approximation of the logsum operation, outputting the final result. It performs a multiplication (MUL) and an addition (ADD) between *m*_*diff*_ and *n*_1,?_ and *n*_2,?_, respectively. The final operation is an addition between *m*_*X*_ and *f*_PLA_(*m*), thus completing the computation of the logsum.

This is the equation implemented in our approach. Fig. 2 contains a circuit diagram showing how the logsum is computed using GC, with *N* = 2 inputs and *k* = 4 look-up table entries.

This diagram raises several implementation concerns. One of the most relevant is the number of bits (ℓ)—how it increases with each computation and what can be done to keep it as small as possible, since more bits mean more computational costs and consequently longer execution times. The operations where the number of bits of the output is larger than the number of bits of the inputs are ADD (1 extra bit), SUB (1 extra bit) and MUL (ℓ extra bits), so the other operations will be ignored in this analysis. Regarding the SUB operation, since the output will always be a negative number, the sign bit is uninformative, and therefore can be discarded. As for the MUL and the first ADD operations, the ℓ least significant bits of *f*_PLA_(*m*) need to be truncated, as otherwise one may end up with a scaling factor different from the one of *m*_*X*_ (recall that all numbers are being represented with fixed-point instead of floating-point). We also remove the most significant bit, as preliminary experiments suggest that it never contains any useful information, i.e., it is always 0. On the last ADD we do not remove any bits, thus increasing the total number of bits by 1 every time the logsum operation is computed.

A second concern is the choice between the SQ and RQ approaches to the PLA. Both of them were implemented following the diagram in Fig. 2, but whereas the RQ approach requires all the blocks, for the SQ approach the MUL and the first ADD block can be ignored, since in this situation *n*_1,?_ = 0. This also means that for the SQ approach there is no need to reduce the number of bits of the output.

#### Logsum of any *N* values

**Fig 3:**
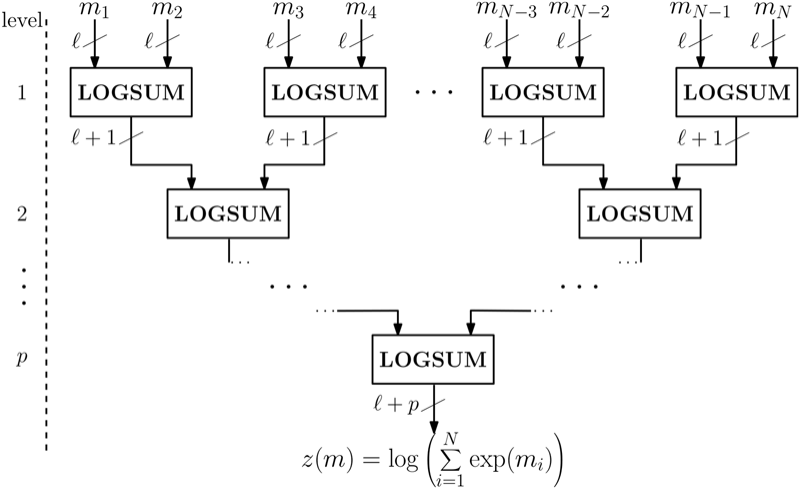
Circuit diagram for the logsum operation for any **N** inputs. The logsum of *N* elements is computed using a hierarchical structure, computing the logsum of two elements at a time. After each level the number of bits required to represent the output increases by 1. If the inputs are represented by ℓ bits, the output will be represented by ℓ+*p* bits, with *p* = ⌈log_2_ *N*⌉.

The Garbled Circuit described previously implements the logsum operation for the simplest case of *N* = 2 input values. Here we present the generalization of this methods to any given *N*. Let us start by recalling the original formulation of the logsum in Equation 3, which for *N* = 4 can be written as

$$\begin{array}{ccc} z(m) & = & \log\left(\exp\left(m_{1}\right)+\exp\left(m_{2}\right)+\exp\left(m_{3}\right)+\exp\left(m_{4}\right)\right)\\ & = & \log\left(e^{\log\left(\exp\left(m_{1}\right)+\exp\left(m_{2}\right)\right)}+e^{\log\left(\exp\left(m_{3}\right)+\exp\left(m_{4}\right)\right)}\right)\\ & = & \log\left(e^{z(m\prime )}+e^{z(m\prime \prime )}\right), \end{array}$$ (6)

with *m* ^′^ = (*m*_1_,*m*_2_) and *m* ^′′^ = (*m*_3_,*m*_4_). This means that the logsum for *N* = 4 input values can be obtained by computing two logsum operations, each of two input values. This process can be repeated as many times as needed, leading to a hierarchical tree structure for computing the logsum operation, as illustrated in Fig. 3. With this structure, the difference in the number of bits between the output and the inputs is *p* = ⌈log_2_ *N*⌉.

## Results and Discussion

In order to evaluate the performance of our implementation of the logsum operation, two sets of experiments were performed. The first set was designed to analyze the effect in the absolute error of changing the number of bits of the input (ℓ) and the number of entries on the look-up table (*k*) when computing the logsum of *N* = 2 input values. The second set was designed to analyze the effect in terms of absolute error and execution time of computing the logsum of input arrays of different sizes *N*, for selected values of ℓ and *k*. We considered two different GC toolkits: the one from the VIPP group [18] and the JustGarble toolkit [19]. Both toolkits implement many techniques mentioned earlier for improving the efficiency of GC, namely pre-computing OT, using the permute technique for gate decryption, free XOR gate evaluation, etc. However, the VIPP toolkit was designed for performing tasks where only a very small number of gates (i.e. a few thousand) are required and not-so-small execution times (i.e. a few seconds) are acceptable. Examples of such tasks include the approaches for iris matching and ECG signal quality evaluation, described in [39] and [20], respectively. Therefore, the VIPP toolkit was not optimized in terms of circuit file size and memory usage. As for the JustGarble toolkit, it was designed in order to both maximize the number of gates that can be used in a single circuit and minimize execution time and memory usage. This means that special care was taken in terms of memory management during the software implementation phase, which has a profound impact in terms of the overall execution time. The advantage of the VIPP toolkit is that, unlike the JustGarble toolkit, it comes with efficient basic block designs in terms of the amount of non-XOR gates for most of the operations we require for computing the logsum. The errors obtained in all experiments are always the same regardless of the GC toolkit considered; the difference occurs only when the execution times are analyzed.

The results obtained in terms of error in the first experiment for both the step quantization (SQ) and the ramp quantization (RQ) approaches are presented in Tables 3 and 4, respectively. In every cell of both tables, the first and second rows contain the mean and maximum absolute error with respect to the true value of the logsum, respectively. Each experiment was performed by generating one million uniformly distributed pairs of numbers and computing the logsum over each pair, both with our approach and the exact equation. Analyzing the results for the SQ approach, we notice that the error obtained depends much less on ℓ than on *k*, as increasing the number of bits beyond ℓ = 12 does not visibly reduce the error, but increasing *k* leads to a significant reduction of the error, specially for larger values of ℓ. This is expected, since the maximum error in this approach is directly governed by the size of the steps, which is much greater than the precision of the values. In the RQ approach, both increasing ℓ and *k* leads to large reductions in the error. In some cases, even small changes in the parameters result in differences of up to one order of magnitude. Although the error obtained using the SQ approach may be expected to always be larger than the one obtained using the RQ approach, for ℓ = 8 this is not the case. The reason may be that the effect of removing the least significant bits after the MUL block is more significant if there is a small number of bits to start with.

**Table 3:** Absolute error for different values of **ℓ** and **k**; step quantization (SQ) approach.

	*k*		
ℓ	32	64	128
8	1.17×10^−2^	1.01×10^−2^	1.03×10^−2^
	(5.49×10^−2^)	(3.74×10^−2^)	(4.05×10^−2^)
12	0.53×10^−2^	0.28×10^−2^	0.15×10^−2^
	(2.74×10^−2^)	(1.52×10^−2^)	(0.89×10^−2^)
16	0.52×10^−2^	0.27×10^−2^	0.13×10^−2^
	(2.68×10^−2^)	(1.46×10^−2^)	(0.83×10^−2^)
24	0.52×10^−2^	0.27×10^−2^	0.13×10^−2^
	(2.68×10^−2^)	(1.45×10^−2^)	(0.82×10^−2^)
32	0.52×10^−2^	0.27×10^−2^	0.13×10^−2^
	(2.68×10^−2^)	(1.45×10^−2^)	(0.82×10^−2^)

ℓ corresponds to the number of bits representing the inputs and *k* is the number of entries of the look-up table for the piecewise linear approximation. In each cell, the first and second rows contain the mean and maximum absolute error with respect to the true value of the logsum, respectively.

**Table 4:** Absolute error for different values of **ℓ** and **k**; ramp quantization (RQ) approach.

	*k*		
ℓ	32	64	128
8	1.69×10^−2^	1.66×10^−2^	1.57×10^−2^
	(5.02×10^−2^)	(5.42×10^−2^)	(5.01×10^−2^)
12	1.10×10^−3^	1.01×10^−3^	1.02×10^−3^
	(3.58×10^−3^)	(3.33×10^−3^)	(3.50×10^−3^)
16	1.45×10^−4^	0.77×10^−4^	0.68×10^−4^
	(4.40×10^−4^)	(2.66×10^−4^)	(2.30×10^−4^)
24	1.20×10^−4^	0.30×10^−4^	0.08×10^−4^
	(2.54×10^−4^)	(0.66×10^−4^)	(0.20×10^−4^)
32	1.20×10^−4^	0.30×10^−4^	0.07×10^−4^
	(2.54×10^−4^)	(0.66×10^−4^)	(0.19×10^−4^)

ℓ corresponds to the number of bits representing the inputs and *k* is the number of entries of the look-up table for the piecewise linear approximation. In each cell, the first and second rows contain the mean and maximum absolute error with respect to the true value of the logsum, respectively.

The results obtained in terms of error in the second experiment for the SQ and RQ approaches are presented in Tables 5 and 6, respectively. Similarly to the previous experiment, in every cell of all the tables the first and second rows contain the mean and maximum absolute error regarding the logsum, respectively, and one million uniformly distributed arrays of size *N* were considered. Regarding the SQ approach, we observe that increasing *N* leads to only slightly increasing the error accumulated by successively computing the logsum, meaning that this technique scales well with *N* for all values of ℓ and *k*. Observing the results for the RQ approach, we notice that it only scales efficiently with *N* for larger values of ℓ and *k*. However, since this is the most interesting situation because it is the one where smaller errors are obtained, scenarios with small ℓ and *k* may be ignored.

**Table 5:** Absolute error for selected values of **N**, **ℓ** and **k**; step quantization (SQ) approach.

*N*	8	32	128	512
ℓ = 8, *k* = 32	0.17×10^−1^	0.24×10^−1^	0.41×10^−1^	0.66×10^−1^
	(1.00×10^−1^)	(1.14×10^−1^)	(1.22×10^−1^)	(1.41×10^−1^)
ℓ = 12, *k* = 128	0.27×10^−2^	0.35×10^−2^	0.40×10^−2^	0.46×10^−2^
	(1.69×10^−2^)	(1.80×10^−2^)	(2.13×10^−2^)	(2.32×10^−2^)
ℓ = 24, *k* = 128	0.26×10^−2^	0.32×10^−2^	0.35×10^−2^	0.37×10^−2^
	(1.39×10^−2^)	(1.79×10^−2^)	(1.84×10^−2^)	(2.06×10^−2^)

*N* corresponds to the number of inputs for the logsum, ℓ corresponds to the number of bits representing the inputs and *k* is the number of entries of the look-up table for the piecewise linear approximation. In each cell, the first and second rows contain the mean and maximum absolute error with respect to the true value of the logsum, respectively.

**Table 6:** Absolute error for selected values of **N**, **ℓ** and **k**; ramp quantization (RQ) approach.

*N*	8	32	128	512
ℓ = 8, *k* = 32	0.48×10^−1^	0.79×10^−1^	1.11×10^−1^	1.43×10^−1^
	(1.12×10^−1^)	(1.37×10^−1^)	(1.66×10^−1^)	(1.90×10^−1^)
ℓ = 12, *k* = 128	0.28×10^−2^	0.48×10^−2^	0.68×10^−2^	0.89×10^−2^
	(0.69×10^−2^)	(0.83×10^−2^)	(1.01×10^−2^)	(1.20×10^−2^)
ℓ = 24, *k* = 128	1.10×10^−5^	1.40×10^−5^	1.80×10^−5^	2.40×10^−5^
	(4.50×10^−5^)	(5.40×10^−5^)	(6.50×10^−5^)	(7.00×10^−5^)

*N* corresponds to the number of inputs for the logsum, ℓ corresponds to the number of bits representing the inputs and *k* is the number of entries of the look-up table for the piecewise linear approximation. In each cell, the first and second rows contain the mean and maximum absolute error with respect to the true value of the logsum, respectively.

We now analyze the execution time of our approach using GC. Currently, it takes a few nanoseconds for a microprocessor to compute a logsum over *N* = 2 elements. Because of the additional operations required by GC (encryption/decryption, shuffling, etc.), our approach requires substantially larger amounts of time to perform the logsum compared to the non-private counterpart. Nevertheless, for many real-time applications, this approach is still fast enough. Taking advantage of all techniques for efficient implementation of GC, all steps but the actual circuit evaluation can be performed offline; therefore, we only present the execution times for that particular step. We confirmed experimentally that if these techniques were not considered, the overall execution times would increase at least by one order of magnitude.

**Fig 4:**
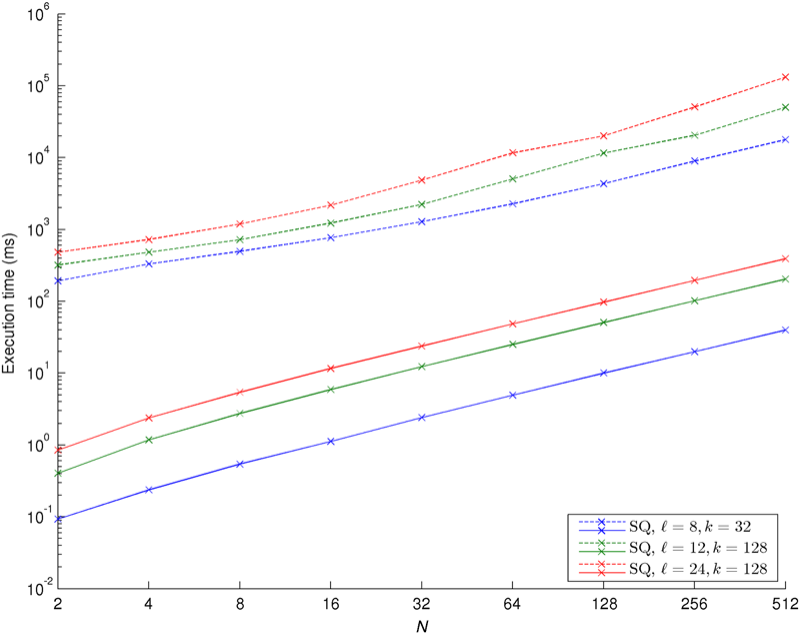
Execution time for selected values of **N**, **ℓ** and **k**, step quantization (SQ) approach, when an Intel Core i7-3630QM CPU @ 2.40GHz with a 6MB L3 cache memory and an 8GB DDR3 RAM memory is considered. *N* corresponds to the number of inputs for the logsum, ℓ corresponds to the number of bits representing the inputs and *k* is the number of entries of the look-up table for the piecewise linear approximation. The dashed lines correspond to the experiments performed with the VIPP toolkit; the solid lines correspond to the experiments performed with the JustGarble toolkit.

**Fig 5:**
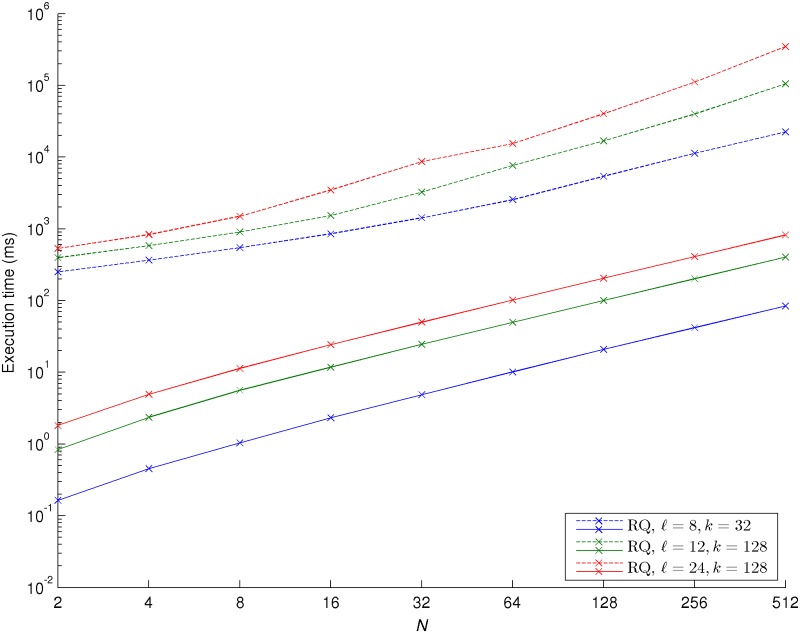
Execution time for selected values of **N**, **ℓ** and **k**, ramp quantization (RQ) approach, when an Intel Core i7-3630QM CPU @ 2.40GHz with a 6MB L3 cache memory and an 8GB DDR3 RAM memory is considered. *N* corresponds to the number of inputs for the logsum, ℓ corresponds to the number of bits representing the inputs and *k* is the number of entries of the look-up table for the piecewise linear approximation. The dashed lines correspond to the experiments performed with the VIPP toolkit; the solid lines correspond to the experiments performed with the JustGarble toolkit.

We considered two different processor and memory settings for our experiments: 1) an Intel Core i7-3630QM CPU @ 2.40GHz with a 6MB L3 cache memory and an 8GB DDR3 RAM memory and 2) an Intel Xeon E5530 CPU @ 2.40GHz with a 8MB L3 cache memory and a 48GB DDR3 RAM memory. The results obtained for the SQ and RQ approaches for the first setting are presented in Figs. 4 and 5, respectively. The execution times obtained for both GC toolkits when the second setting was considered are around 10% to 15% slower than the ones from the first setting, but have otherwise a very similar behavior to them. Therefore, and in order to avoid overloading this section, we decided not to reproduce them. Each of the results was obtained by averaging the execution time of one thousand consecutive runs of the logsum algorithm. As expected, for both GC toolkits and using the same values of ℓ and *k*, the RQ approach takes longer than the SQ approach to compute a logsum. We observe that for the VIPP toolkit there is a near quadratic increase of the execution time with increasing values of *N*, probably a consequence of the successively larger circuits that need to be loaded into memory. We also notice that for the JustGarble toolkit there is a constant linear relationship between how long it takes to compute the logsum and the number of inputs for all values of ℓ and *k*, meaning that our approach also scales nicely with *N* regarding the execution time. Finally and more importantly, the JustGarble toolkit is consistently two or three orders of magnitude faster than the VIPP toolkit, definitely showing that when larger garbled circuits are considered, it is extremely important to use a toolkit specially designed for optimal memory management.

## Conclusions

This paper presents a technique for computing the logsum operation using Garbled Circuits. We describe a simple technique that approximates the logsum with a piecewise linear approximation, which is then expressed in the form of a garbled circuit that can be computed efficiently, while maintaining the privacy of its inputs. Furthermore, even within this format, unlike previous approaches, the solution is scalable, in the sense that the accuracy of the approximation can be traded off for computation time by increasing the parameters ℓ and *k*. The SQ and RQ options offer the following choice—the SQ approach can be used for fast computation where the underlying problem can tolerate lower precision, and RQ can be used where greater precision is required. Even with the increased computation, this method is still much faster than previously proposed homomorphic encryption approaches. We evaluated two different GC toolkits designed for distinctive ends, and concluded that memory management should be a prime concern when implementing a general purpose GC toolkit.

The logsum operation is an important cornerstone for many signal processing applications. The technique proposed here presents an ideal primitive to be incorporated in the implementation of such applications in a privacy-preserving scenarios.
